# Supplementary material for: Causal Relationship between Aging and Anorexia Nervosa: A White-Matter-Microstructure-Mediated Mendelian Randomization Analysis
Source: Biomedicines. 2024 Aug 16;12(8):1874. doi: 10.3390/biomedicines12081874 (PMC11351342; doi:10.3390/biomedicines12081874)
Supplement: Supplementary file 1 [file biomedicines-12-01874-s001.zip › Supplementary Figure/Figure S4/Singe SNP plot of MR analysis for TL to ubm-b-2117.pdf]

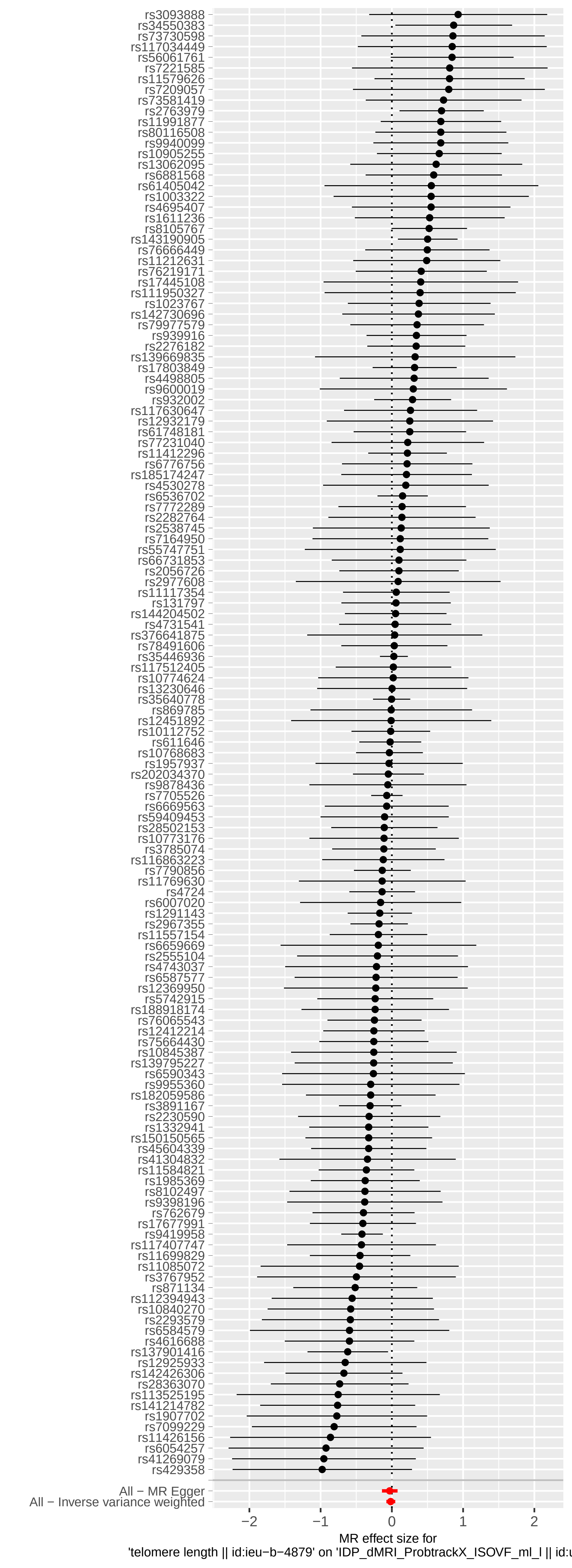
rs3093888
rs34550383
rs73730598
rs117034449
rs56061761
rs7221585
rs11579626
rs7209057
rs73581419
rs2763979
rs11991877
rs80116508
rs9940099
rs10905255
rs13062095
rs6881568
rs61405042
rs1003322
rs4695407
rs1611236
rs8105767
rs143190905
rs76666449
rs11212631
rs76219171
rs17445108
rs111950327
rs1023767
rs142730696
rs79977579
rs939916
rs2276182
rs139669835
rs17803849
rs4498805
rs9600019
rs932002
rs117630647
rs12932179
rs61748181
rs77231040
rs11412296
rs6776756
rs185174247
rs4530278
rs6536702
rs7772289
rs2282764
rs2538745
rs7164950
rs55747751
rs66731853
rs2056726
rs2977608
rs11117354
rs131797
rs144204502
rs4731541
rs376641875
rs78491606
rs35446936
rs117512405
rs10774624
rs13230646
rs35640778
rs869785
rs12451892
rs10112752
rs611646
rs10768683
rs1957937
rs202034370
rs9878436
rs7705526
rs6669563
rs59409453
rs28502153
rs10773176
rs3785074
rs116863223
rs7790856
rs11769630
rs4724
rs6007020
rs1291143
rs2967355
rs11557154
rs6659669
rs2555104
rs4743037
rs6587577
rs12369950
rs5742915
rs188918174
rs76065543
rs12412214
rs75664430
rs10845387
rs139795227
rs6590343
rs9955360
rs182059586
rs3891167
rs2230590
rs1332941
rs150150565
rs45604339
rs41304832
rs11584821
rs1985369
rs8102497
rs9398196
rs762679
rs17677991
rs9419958
rs117407747
rs11699829
rs11085072
rs3767952
rs871134
rs112394943
rs10840270
rs2293579
rs6584579
rs4616688
rs137901416
rs12925933
rs142426306
rs28363070
rs113525195
rs141214782
rs1907702
rs7099229
rs11426156
rs6054257
rs41269079
rs429358
All − MR Egger
All − Inverse variance weighted
−2
−1
0
1
2
MR effect size for
'telomere length || id:ieu−b−4879' on 'IDP_dMRI_ProbtrackX_ISOVF_ml_l || id:u
